# Microplastics in Stormwater: Sampling and Methodology Challenges

**DOI:** 10.3390/toxics13060502

**Published:** 2025-06-14

**Authors:** Andres Sanchez Garcia, Huayun Zhou, Cesar Gomez-Avila, Tariq Hussain, Aryan Roghani, Danny Reible, Balaji Anandha Rao

**Affiliations:** 1 Department of Chemical Engineering, Texas Tech University, Lubbock, TX 79409, USA; andres.sanchez@ttu.edu (A.S.G.);; 2 Department of Civil, Environmental & Construction Engineering, Texas Tech University, Lubbock, TX 79409, USA; 3 Haley & Aldrich, Portland, OR 97239, USA; 4 Department of Biomedical Engineering, University of Texas at Austin, Austin, TX 78712, USA

**Keywords:** microplastics, stormwater, Nile red, fluorescence microscopy, Raman spectroscopy

## Abstract

Stormwater runoff is a significant source of microplastics to surface water. This study addresses challenges in the sampling, treatment, and characterization of microplastics in existing stormwater control measures across various regions in the United States. Stormwater sediment samples were collected via traditional stormwater sampling approaches for particulate and inorganic contamination with portable automatic samplers, analyzed using visible and fluorescence microscopy with Nile red as a selective stain, and subsequently confirmed through Raman spectroscopy. The inclusion of laboratory and field blanks enabled the identification of contamination at key steps during sample handling. The results reveal that the filtration process is a significant source of laboratory contamination, while the sampling process itself could be a primary contributor to overall sample contamination. Additionally, it was found that using green fluorescence as the sole emission wavelength may underestimate MP quantities, as some particles emit fluorescence exclusively in the red spectrum. Raman analysis revealed interferences caused by pigments and additives in plastics, along with challenges evaluating particles in the low micron range (≤10 microns), which complicates a comprehensive analysis. The findings of this study emphasize the importance of implementing strong quality assurance and control measures when assessing the levels of microplastics in the environment, including sample collection, processing, and analysis.

## 1. Introduction

Environmental microplastics (MPs), generally defined as plastic particles smaller than 5 mm [1], comprise a heterogeneous assemblage of particles that vary in size, shape, color, density, chemical composition, and other characteristics [2]. MPs can be broadly classified based on their source, with primary MPs considered as precursors from the direct discharge of manufactured plastic products, including those used in cleaning and personal care products [2,3,4,5], while fragments with irregular shapes generated from abrasion of larger plastic objects are considered secondary MPs. Tire and road wear particles (TRWPs) that are of relevance in stormwater matrices are generally lumped together with MPs for characterization and assessment [6,7,8].

The most frequently reported MPs in stormwater are polyethylene (PE), polypropylene (PP), polystyrene (PS), polyvinyl chloride (PVC), polyurethane (PUR), and polyethylene terephthalate (PET) [9,10,11]. Both MPs and TRWPs can be transported by wind or water runoff, along with debris on the ground, into stormwater drains [12,13] and eventually settle in receiving environments (e.g., sediments), where they can impart toxic effects [13,14,15]. Seabirds, crustaceans, fish, and other marine biota can ingest deposited MPs, resulting in reduced food intake [16]. In addition, MPs can adsorb chemical contaminants like persistent organic pollutants (polycyclic aromatic hydrocarbons PAHs, polychlorinated biphenyls PCBs), antibiotics, organochlorine pesticides (OCPs), dichloro-diphenyl-trichloroethane (DDT) and hexachlorocyclohexane (HCH) insecticides, and heavy metals [17,18] via partitioning to their surfaces, facilitating concentrations at levels several orders of magnitude higher than those in the surroundings [19]. MPs can not only sorb and bind toxic pollutants from the environment but they can often release these compounds back into the environment. Various additives that are used as processing compounds in relatively high concentrations have been shown to be released during their disintegration [17].

Identifying and quantifying MPs often involves examining physio-chemical characteristics, including polymer type, shape, size, and color [4,14,17,20,21]. Although no validated method currently exists for the collection and preparation of stormwater samples, stormwater MP assessments typically include stormwater sampling followed by MP separation and identification. The sampled stormwater is sieved or filtered, and the accumulated stormwater particles typically undergo selective chemical oxidation (e.g., hydrogen peroxide oxidation) to remove background organic particles [2,22]. Visual and fluorescence microscopy are commonly used as preliminary techniques to estimate particle counts and examine physical attributes like size, shape, and color, as they are simple and accessible; however, these methods have the disadvantage of being susceptible to inaccuracies as they rely on subjective evaluation [2,4,10,21]. By adding lipophilic dyes, fluorescence microscopy enhances MP visualization by selectively staining synthetic polymers, allowing them to be distinguished from minerals. However, it may still produce false positives in the presence of bio-polymers (e.g., lignin) recalcitrant to chemical digestion [23,24,25,26]. While confirmatory techniques like Raman and Fourier transform infrared (FTIR) spectroscopy are essential for determining MP polymer type [27,28,29,30,31], they have limitations, including time-consuming sample preparation, low spatial resolution, and challenges with highly absorptive materials such as TRWPs [32,33].

Differences between the sampling, laboratory processing, and analysis techniques adopted in other studies have led to a wide variety of results [21,34]. Additionally, selecting an adequate process for organic matter removal, including reagents and temperatures, is crucial to minimize the loss of constituent microplastics due to damaging [22,35]. Finaly, it has also been established that no single analytical technique will offer a comprehensive characterization of microplastics, as they offer different trade-offs with respect to sensitivity, specificity, and precision [36].

Considering that the use of automatic samplers is a common practice for stormwater sampling [37,38,39,40,41,42,43,44], there is a lack of detailed characterization of MPs, particularly on the suitability of existing sampling techniques. This study focuses on the challenges encountered during the sampling, treatment, and application of the most commonly used screening (visual and fluorescence microscopy) and confirmatory (Raman spectroscopy) techniques for the characterization of MPs in real stormwater systems. Additionally, this study provides a robust pathway for stormwater MP characterization by identifying common areas of concern in the reported methodologies for stormwater MP analysis.

## 2. Materials and Methods

### 2.1. Materials

Polytetrafluoroethylene (PTFE) membrane filters (Millipore Sigma, Burlington, MA, USA, 0.45 μm pore size) were used to filter the collected stormwater samples with a glass vacuum filtration system. Glass vials (40 mL) (Environmental Express, Charleston, SC, USA, Level I quality assured) were employed for the digestion process, which used hydrogen peroxide (Fisher Scientific, Waltham, MA, USA, 30% *v/v*). Aluminum oxide (AlOx) membrane filters (Whatman, Maidstone, UK, 0.1 μm pore size) were used to filter the samples post-digestion, with assistance of a vacuum filtration system. Deionized (DI) water from a GenPure Pro UV (Thermo Scientific, Waltham, MA, USA) was used to rinse all material before being used and for filtration. Nile red (NR) dye (Thermo Fisher, purity ≥ 95%) was diluted in Koptec, 190 proof ethanol (Decon Labs, King of Prussia, PA, USA, purity ≥ 95%) for the fluorescent tagging of the samples.

### 2.2. Stormwater Samples Collection

An existing standard sampling technique, developed for the evaluation of particulate and inorganic contaminants typically present in stormwater [37,38,39,41], was applied to evaluate the MPs. Runoff samples from seven urban stormwater control measures (SCMs) and mixed industrial–commercial land use were collected across the U.S. (Table 1). Three SCMs located in Bremerton, Washington (B1, B2, and B3), three SCMs in San Diego, California (SD1, SD2s and SD3), and one SCM in Lubbock, Texas (TX). Samples were collected using model 3700 portable automatic samplers in conjunction with Signature Flowmeters and 674 rain gauges (all from TELEDYNE ISCO, Lincoln, NE, USA) or Sigma 900 Max samplers (HACH, Ames, IA, USA). Existing Sigma 900 samplers were used at the SD1 location; all the other sites were sampled with 3700 samplers. These samplers were positioned at both the inlet and outlet of each SCM to monitor its performance, except at TX, where the samplers were located at each of the three inlets to the retention pond. The samplers were set to automatically trigger when certain conditions for rainfall and water were met. The model 3700 samplers were programmed to collect a composite sample of 10 L, consisting of 20 subsamples of 500 mL each at 5 min intervals after the trigger. The Sigma 900 samplers were programmed to capture a 10 L composite sample consisting of 10 subsamples of 1 L each at intervals of 250 gallons of rain [37]. Pre-cleaned (washed with phosphate-free detergent and rinsed with DI water) 10 L glass bottles were transported to each sampling site and placed inside the samplers while the bottle lids were kept separately within a clean plastic bag. Vinyl tubing was used to capture runoff for all sampler systems. Following a storm event, the sampling program was reviewed to confirm collection times and volumes. Specific location IDs were written on the lids of glass bottles, and the samples were placed in coolers for transportation to the lab. Finally, samples were split using a Dekaport sample splitter (Geotech Environmental Equipment, Denver, CO, USA) into ten high-density polyethylene (HDPE) bottles of 1 L each. Nine of the ten bottles were used for trace metals analysis only, and those results will be published elsewhere.

### 2.3. Sample Treatment

The sample contained in the 1 L bottle was vacuum-filtered on a PTFE filter. Generous amounts of DI water were used to ensure maximum sample recovery from the bottles. The filter was then placed in an oven and left to dry for 24 h at 60 °C. After the sample was dried, a representative quarter of the PTFE filter was cut and placed into a 40 mL glass vial. The remaining three quarters were used for trace metals analysis only, and those results will be published elsewhere. A modified peroxide oxidation was performed in a fume hood by adding 20 mL of concentrated H_2_O_2_ to the vial and subsequently covering it with aluminum foil that had been punctured to create a small opening in the top to allow evaporation while minimizing potential contamination of the sample from airborne MPs. The vial was then placed in a digestor (Brooks Rand LINX, Seattle, WA, USA) set at 65 °C for a period of 72 h. The volume of H_2_O_2_ was maintained at a constant level (~20 mL) by adding aliquots as needed during the digestion process. After digestion, the content of the vial was filtered through an AlOx filter via a vacuum filtration system and with an abundant amount of DI water to ensure complete transfer. The vacuum filtration system was covered with an aluminum pan to minimize airborne contamination. The AlOx filter was placed on a glass slide inside a Petri dish with the lid and was left to dry at room temperature for at least 24 h.

### 2.4. MP Characterization

#### 2.4.1. Visual Analysis

**Figure 1:**
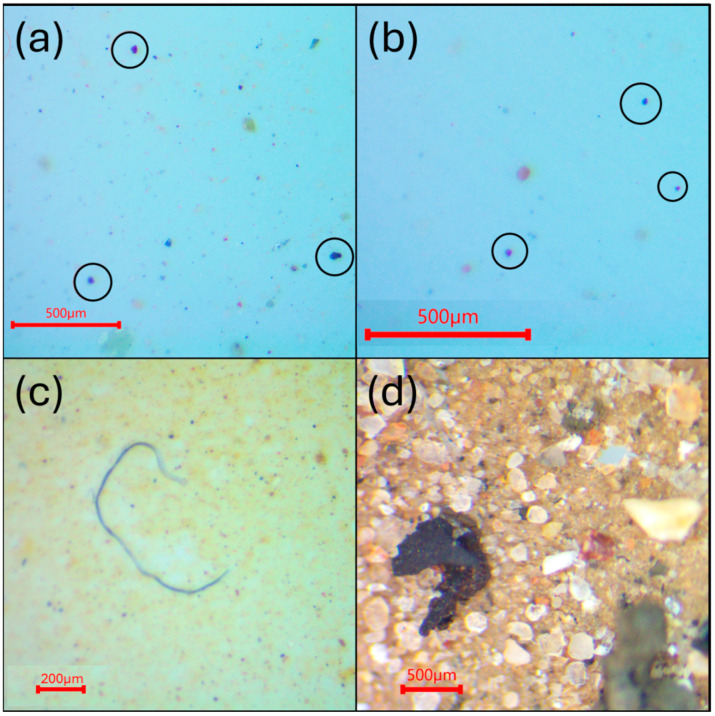
Images from the visual microscopy. Examples of fragments (objects circled in (**a**)), pellets (objects circled in (**b**)), a fiber (**c**), and a TRWP (**d**).

Visual analysis of the particles on the AlOx filters was conducted with a trinocular stereo zoom microscope (AmScope, Irvine, CA, USA) with a ring light and mount camera at 20× magnification. A full sweep of the filter was performed starting from the bottom left of the filter and reading from left to right, then moving up ‘one row’ and reading from right to left. This process was repeated until the whole filter was read. MP candidates were counted and classified based on color (e.g., blue, black, red) and categorized into different morphologies based on their shape (fibers, fragments, or pellets). Examples of the morphology of each class are shown in Figure 1. In general, elongated filaments were considered fibers, particles with irregular shapes were classified as fragments, and pellets had a well-defined round shape; black particles with irregular form and rubber-like texture were categorized as TRWPs. Particles with a mineral appearance (e.g., sediments, rocks or sand) were ignored during the counting. Particle size distribution analysis was performed using ImageJ software (version 1.54p).

The concentration of MPs was estimated by normalizing the number of microplastics counted on each filter and dividing by the sample volume, as indicated in Equation (1):

(1) $$MPs\;concentration\;\left[\frac{MPs}{L}\right]=\frac{{MP}_{f}\cdot 4}{V}\cdot 1000$$

where

*MPs* is the number of microplastics counted on the AlOx filter.

*V* is the volume of the sample that was filtered [mL].

4 is a conversion factor to consider all the quarters from the original PTFE filter from where the sample was taken.

1000 is the conversion factor from mL to L of sample.

#### 2.4.2. Fluorescence Analysis

A Nile red solution (1 mg of NR/mL of ethanol) [26] was used to stain particles by adding it dropwise onto the filters while ensuring full coverage of the particles and leaving the filter to dry at room temperature overnight inside a glass Petri dish with the lid on. Once dry, the sample was analyzed with a BX41 fluorescence microscope with a trinocular head at 4× magnification (Olympus, Center Valley, PA, USA). Identical to the visual analysis, the sample filter was completely swept starting from bottom left, and the estimated concentration was also calculated using Equation (1). MP candidates were counted and classified based only on shape, as color is not distinguishable in fluorescence. A green fluorescent protein (GFP) filter set (excitation: 457–487, emission: 502–538 nm) and a Texas red (TxRed) filter set (excitation: 542–582, emission: 604–644 nm) were used to cover most of the NR excitation/emission ranges (excitation: 400–600, emission: 525–750 nm). Particles that showed fluorescence on both filter sets were not counted during the GFP sweep to avoid double counting.

#### 2.4.3. Raman Analysis

Samples were analyzed using a SENTERRA dispersive Raman microspectrometer (Bruker, Billerica, MA, USA) to characterize the chemical identity of the MP candidates found in the previous analysis. TRWPs could also be confirmed with this technique by targeting carbon black (CB) during the analysis, as CB represents 20 to 35% by weight of a passenger car tire [32]. All spectrums were acquired using either a 532 nm or a 785 nm laser, with an acquisition time of 10 s and a laser power of 2 mW using a 20× magnification lens. Spectrum identification was performed using the tool ‘Quick Compare’ that comes with the software OPUS (Bruker, version 8.5.29 (64 bit)), as well as Open Specy (accessed on 3 June 2025).

### 2.5. Quality Assurance

Laboratory practices were implemented to minimize contamination from airborne MPs. All materials used from sample pretreatment to Raman analysis were rinsed with abundant DI water right before being used. Lab benches were covered with aluminum foil. A single sample was handled at a time and was kept capped when not being used.

Method blanks (MBs), which followed the same processing steps from isolation to Raman analysis, were used to determine the level of contamination from sample handling. One quarter of a clean, new PTFE filter was cut and placed into a 40 mL glass vial. The filter underwent the modified peroxide oxidation in a fume hood by adding 20 mL of concentrated H_2_O_2_ to the vial and subsequently covering it with aluminum foil that had been punctured to allow evaporation while minimizing the potential contamination of the sample from airborne MPs. The vial was then placed in the digestor at 65 °C for a period of 72 h. The volume of H_2_O_2_ was maintained at a constant level (~20 mL) during the digestion process. After digestion, the content of the vial was filtered through an AlOx filter using the vacuum filtration system and an abundant amount of DI water to ensure complete transfer. The vacuum filtration system was covered with an aluminum pan to minimize airborne contamination. The AlOx filter was placed on a glass slide inside a Petri dish with the lid on and left to dry at room temperature for at least 24 h. The sample was then analyzed by visual and fluorescence microscopy. This process was repeated to create replicates.

A field blank (FB) was prepared to assess potential contamination introduced by sampling materials and activities, including sample transportation. The field blank consisted of 500 mL of DI water that was pumped through one of the 3700 portable automatic samplers at the TX location. The system was initially rinsed with 1 L of DI water that was passed through the sampling system; subsequently, the 500 mL of DI water was passed through and collected in a 1 L glass bottle. The sample contained in the 1 L bottle was vacuum-filtered on a PTFE filter. A generous amount of DI water was used to ensure complete recovery from the bottle. The filter was then placed in an oven and left to dry for 24 h at 60 °C. After the filter was dried, three quarters of it were cut and placed into three individual 40 mL glass vials. The filters underwent the modified peroxide oxidation in a fume hood by adding 20 mL of concentrated H_2_O_2_ to each vial and subsequently covering them with aluminum foil that had been punctured to allow evaporation while minimizing potential contamination from airborne MPs. The vials were then placed in the digestor at 65 °C for a period of 72 h. The volume of H_2_O_2_ was maintained at a constant level (~20 mL) during the digestion process. After digestion, the content of the vials was filtered through AlOx filters using a vacuum filtration system and an abundant amount of DI water to ensure complete transfer. The vacuum filtration system was covered with an aluminum pan to minimize airborne contamination. The AlOx filters were placed on glass slides inside Petri dishes with lids and left to dry at room temperature for at least 24 h. The filters were then analyzed by visual and fluorescence microscopy.

Additional blanks from key treatment steps were prepared. Four different treatment levels were defined: Level 1 (Filter + Filtration + Digestion + Sampling), which consisted of the field blanks previously mentioned; Level 2 (Filter + Filtration + Digestion), consisting of the method blanks previously mentioned; Level 3 (Filter + Filtration), which consisted of filters that were exposed to the vacuum filtration system to mimic the filtration step; and Level 4 (Filter), which consisted of analyzing a new clean filter with no additional treatment (e.g., not digested nor exposed to vacuum filtration). The four treatment levels were compared with a combination of a *t*-test, analysis of variance (ANOVA), and Tukey’s Honestly Significant Difference (HSD) test to identify the major sources of contamination in the blanks. The analysis was performed for results from visual and fluorescence microscopy. The statistical tests were performed in JMP software (version 18.2.2 (785088)) at a significance level of α = 0.05. Furthermore, the effects of rinsing materials prior to their use (Figure S1), filtering the DI water and the hydrogen peroxide, and the fluorescence of certain plastics under different filter sets were also evaluated.

## 3. Results

### 3.1. Visual Analysis

#### 3.1.1. Stormwater Samples

**Figure 2:**
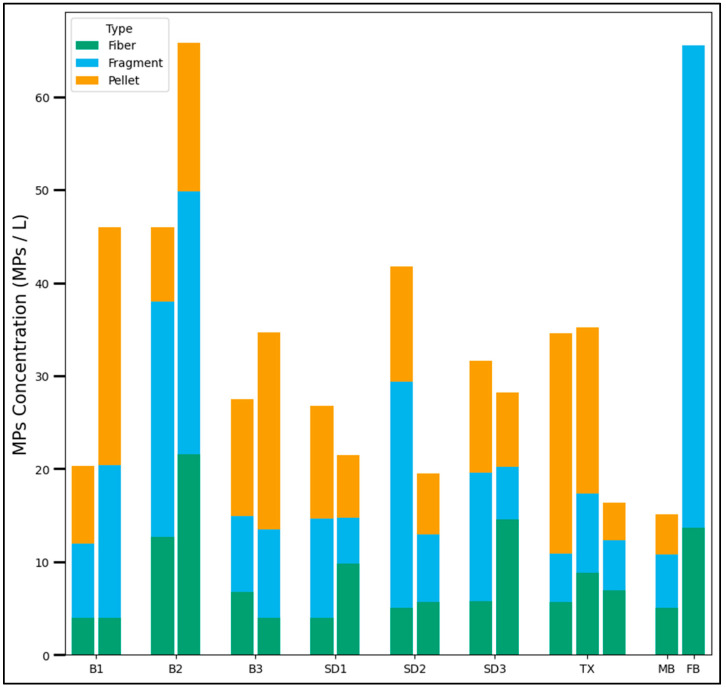
Results from visual analysis for each site. Inlet (left bar) vs. Outlet (right bar) of the corresponding SCM is displayed for each location, except for TX, where Inlet 1, Inlet 2, and Inlet 3 are compared.

The estimated concentration of MPs, as well as the distribution of MPs in terms of shape, for every site and rain event sampled are presented in Figure 2, where every pair of bars are the geometric mean of the concentration for all rain events in the inlet and outlet concentrations for the specific location. The median particle size for the Bremerton and San Diego samples was 40.5 and 46.7 µm, respectively, while the median for the Texas samples was smaller (25.2 µm). The estimated concentration in the method blanks and field blanks were 24 ± 9 and 71 ± 13 MPs/L, with median sizes of 22.1 and 46.9 µm, respectively. The median particle size for the laboratory blanks was similar to that found for the Texas samples, while the sizes from the field blank more closely resembled the Bremerton and San Diego samples. Additional information on the particle size distribution can be found in Figure S2. The most predominant morphology for the particles found in the field blanks were black particles, while blue particles and fibers were predominant in the method blanks. Most samples from visual analysis had MP concentrations above the method blanks but below the field blanks (Figure 2). Additional data showing independent rain events (Figure S3), the color-based classification of MPs (Figure S4), and raw particle counting (Table S1) is presented in the Supplementary Material.

#### 3.1.2. Contamination Source Trials

A summary of the Tukey’s HSD test for the visual microscopy analysis is presented in Table 2. The results indicate that the average MP count in filters exposed only to filtration did not differ significantly from those subjected to both digestion and filtration. This suggests that the filtration step is a major source of contamination in laboratory blanks. However, the field blanks (treatment level 1) showed a significant increase in MP presence compared to method blanks.

### 3.2. Fluorescence Microscopy

The results from fluorescence microscopy, tested with Tukey’s HSD test, indicate no significant differences between treatment levels 2 and 3, as shown in Table 3. Field blanks (treatment level 1) continued to show significantly higher MP counts that were consistent with the visual microscopy results. This suggests that the sampling process itself may introduce a considerable number of MP candidates to the actual samples. Contrary to the previous analysis, the untreated filter exhibited significant contamination. This could be attributed to an improved image contrast from fluorescent light emitted against a dark background, enabling the identification of more particles in the samples compared to visual microscopy.

The concentrations of MPs at the system inlets were roughly five times higher when estimated by fluorescence microscopy compared to the concentrations obtained by visual microscopy for most sites, except for the B1 site (20 times higher). At the SCM outlets, an overall increase of four times was observed, with lower variance compared to the inlet results. In contrast, the TX site exhibited differing trends, with two of the three outfalls showing a reduction in the estimated MP concentration. Laboratory and field blanks showed high concentration ratio values that were surpassed only by the B1 site. The elevated concentrations in the field blank compared to the site samples may be attributed to potential degradation and abrasion of the sampling system tubing, as field blanks were sampled several months later. The results are summarized in Table 4.

**Figure 3:**
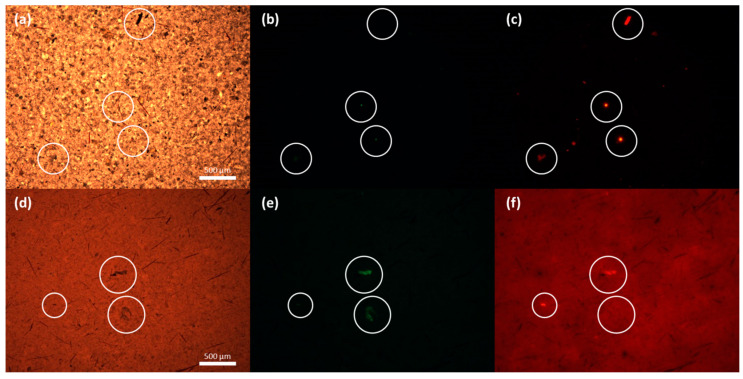
Comparative images from B1 (**a**–**c**) and SD2 (**d**–**f**) analyzed by fluorescence microscope through BF (**a**,**d**), GFP (**b**,**e**), and TxRed (**c**,**f**) filter sets. Some MP candidates show fluorescence only in one of the filter sets (either GFP or TxRed), while some others fluoresce in both.

Multiple authors have chosen to study MPs only under the green fluorescence range [26,45,46]. This can lead to an underestimation of potential MP candidates, as certain particles can only be detected under red fluorescence. An example of this finding can be seen in Figure 3, where a B1 site sample and a sample from the SD2 site were analyzed under bright field (BF), GFP, and TxRed filter sets; not all the particles spotted with the GFP filter can be detected using the TxRed filter and vice versa.

### 3.3. Raman Analysis

**Figure 4:**
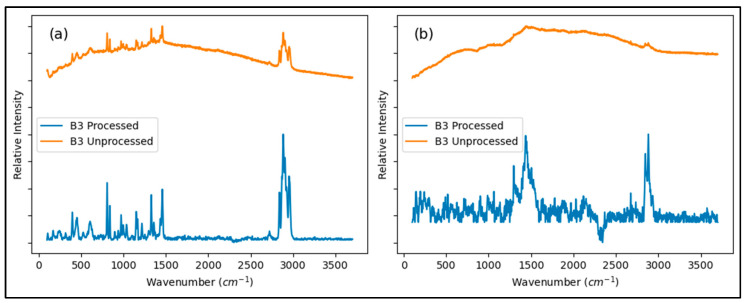
Raman spectra from two different particles present in the B3 inlet sample before and after being processed with OPUS Software. Fluorescence background from the particles is reduced after processing, allowing manual matching with PP (**a**) and HDPE (**b**) standards.

In general, most of the MP candidates identified through visual microscopy were at the lower size range (5–500 microns). While the theoretical size resolution of MPs by Raman microscopy is close to 1 micron, we found particles below 10 microns particularly challenging to characterize due to difficulties focusing on the sample, high background readings, and fluorescence interferences. This limited our ability to properly confirm and characterize the MPs screened from the visual and fluorescence microscopy techniques. In fact, there are very limited studies that have evaluated MPs at sizes bellow 10 microns using Raman microscopy [27]. A paired event (Inlet and Outlet) with the highest presence of MP candidates from SD2 and from B3 were selected for Raman spectroscopy, as well as the field blank. All spectra were processed by performing smoothing, normalizing, and background correction with OPUS software to mitigate background fluorescence (Figure 4). The spectra manipulation was performed after removing spectral points at low wavenumbers (below 100 cm^−1^) to exclude readings near the filter cutoff, enhancing background corrections. In contrast with previous literature reports, the spectra readings using a 735 nm source did not provide identifiable data, while better quality spectra could be acquired, compared, and identified when a 532 nm source was used. A total of 43 MP candidate particles were analyzed. Of these, four were identified by the OPUS software with a matching percentage above 90% (three as TRWPs and one as PET). Four additional particles that could not be matched by the software were manually identified (Table S2) based on the presence of characteristic peaks in the spectra that closely resembled those of the standards (one as a TRWP, two as PP, and one as HDPE). The remaining parts of the spectrum were identified as minerals in Open Specy, with matching percentages below 80%. A summary of the particles successfully identified as MPs can be found in Table 5. The difficulties obtaining good quality spectra can be attributed to strong fluorescence effects caused by the presence of dyes, fillers, and additives in plastics, as previously highlighted by Lenz et al. [47]. Another possibility is that the particles sizes were smaller than the laser beam used, causing the acquisition of signals coming from the background filter instead of from the actual sample.

## 4. Conclusions

Stormwater represents an important source of MPs in receiving environments including coastal waters and sediments. However, there is a lack of information on the suitability of common stormwater sampling, processing, and analytical techniques for characterizing MPs in real stormwater samples. Both the visual and fluorescence microscopy techniques applied in our study indicate that sampling and vacuum filtration steps can significantly contribute to potential MP contamination in stormwater matrices. It is likely that the primary source of contamination for the field blanks comes from the sampling tubes used in the automated samplers. While beyond the scope of this study, the evaluation of the sampling (vinyl) and pump (silicone) tubes used in the automatic sampler for the generation of MP particles is worth exploring. These particles will not be easily distinguishable from those originating from stormwater samples since both visual and fluorescence microscopy techniques are screening tools and do not provide information on polymer chemistry. Nevertheless, our results further emphasize the importance of evaluating both laboratory blanks and field blanks to properly assess the MPs in stormwater. In addition, our research found that a potential underprediction of MPs can occur when using only the green filter for fluorescence microscopy analysis. When red fluorescence was incorporated into the analysis, an increase in MP candidates was detected. The selection of a comprehensive spectral range for fluorescence analysis is necessary to ensure all potential MPs are evaluated. Previous studies have shown that both the solvent and polymer type have a significant influence on the fluorescence characteristics of Nile red and likely depend on the chemical environment’s hydrophobicity and the polarity of the surrounding chemical environment [48,49]. In general, Raman spectroscopy is a common confirmatory technique used in MP characterization and is considered to have better spatial resolution (up to 1 micron) compared to infrared-based techniques. However, we found that the application of Raman microscopy is a labor-intensive process and that there is a practical spatial limitation to particles with sizes smaller than 10 microns. Furthermore, the presence of dyes and additives can contribute to overwhelming background fluorescence, thereby limiting its application. The overall results from this study highlight the need for robust quality assurance and control measures that include a comprehensive evaluation of the sampling, processing, and analytical protocols.

## Figures and Tables

**Table 1:** Description of sampling sites.

Site ID	Site Location	Catchment Type	Type of SCM	No. of Rain Events Sampled
B1	Bremerton area	Industrial	Hydrodynamic separator	3
B2	Bremerton area	Industrial/loading dock	Hydrodynamic separator + media filter	3
B3	Bremerton area	Industrial	Hydrodynamic separator + media filter	2
SD1	San Diego area	Parking lot	Bio-infiltration	3
SD2	San Diego area	Metal fabrication facility + parking lot	Bio-infiltration + media filter	3
SD3	San Diego area	Parking lot	Bio-infiltration	2
TX	Texas	Mixed use	Retention pond	3

**Table 2:** Outcome from the Tukey’s HSD analysis from the visual microscopy results conducted to identify the major contamination source. Three categories were obtained, suggesting the grouping of treatment levels 2 and 3.

Treatment Level	n	Treatment Level Description	Group			Average MP Count	STD
1	3	Filter + Filtration + Digestion + Sampling	A			71	13
2	9	Filter + Filtration + Digestion		B		24	9
3	6	Filter + Filtration		B		16	9
4	6	Filter			C	2	4

**Table 3:** Tukey’s HSD results from the fluorescence microscopy analysis. Here, only two categories were obtained, suggesting the grouping of all treatment levels except for treatment level 1.

Treatment Level	n	Treatment Level Description	Group			Average MP Count	STD
1	3	Filter + Filtration + Digestion + Sampling	A			1219	97
2	9	Filter + Filtration + Digestion		B		214	83
3	3	Filter + Filtration		B	C	107	45
4	3	Filter			C	75	24

**Table 4:** Comparison between total MP concentrations estimated by visual and fluorescence microscopy. The fluorescence to visual concentration ratio is shown to indicate the increase in the concentration estimated by fluorescence when compared to that estimated by visual microscopy.

Stage	Site ID	Visual Microscopy (MPs/L)	Fluorescence Microscopy (MPs/L)	Concentration Ratio (Fluorescence/Visual)
Inlet	B1	21	411	19.6
	B2	51	362	7.10
	B3	60	227	3.78
	SD1	28	138	4.93
	SD2	84	376	4.48
	SD3	36	192	5.33
Outlet	B1	54	89	1.65
	B2	77	207	2.69
	B3	112	316	2.82
	SD1	21	183	8.71
	SD2	22	120	5.45
	SD3	34	217	6.38
Inlet 1	TX	64	41	0.64
Inlet 2	TX	55	197	3.58
Inlet 3	TX	34	33	0.97
Blank	Laboratory	24	214	8.92
	Field	71	1219	17.2

**Table 5:** Summary of Raman results acquired with the 532 nm laser for the three selected locations.

Location	Stage	No. of Particles Analyzed	No. of Particles Identified	Identification Method	MP Identified
SD1	Inlet	14	2	Manually (1) Software (1)	TRWP TRWP
	Outlet	8	2	Software (2)	PET and TWRP
B3	Inlet	9	2	Manually (2)	HDPE and PP
	Outlet	6	1	Software (1)	TWRP
TX	Field Blank	6	1	Manually (1)	PP

## Data Availability

Data sets generated during the current study are available from the corresponding author upon reasonable request.

## Supplementary Materials

The following supporting information can be downloaded at: https://www.mdpi.com/article/10.3390/toxics13060502/s1, Figure S1: Effect of rinsing materials; Figure S2: Particle size distribution for visual microscopy; Figure S3: Results from the visual analysis for all sites; Figure S4: Color-based classification of MPs; Table S1: Raw particle counting; Table S2: Raman sample identification.

## Abbreviations

The following abbreviations are used in this manuscript:

MPs	Microplastics
TRWPs	Tire and road wear particles
PE	Polyethylene
PP	Polypropylene
PS	Polystyrene
PVC	Polyvinyl chloride
PUR	Polyurethane
PET	Polyethylene terephthalate
PAH	Polycyclic aromatic hydrocarbons
PCB	Polychlorinated biphenyls
OCP	Organochlorine pesticides
DDT	Dichloro-diphenyl-trichloroethane
HCH	Hexachlorocyclohexane
FTIR	Fourier transform infrared
PTFE	Polytetrafluoroethylene
AlOx	Aluminum oxide
DI	Deionized
NR	Nile red
SCM	Stormwater control measures
HDPE	High-density polyethylene
GFP	Green fluorescent protein
TxRed	Texas red
CB	Carbon black
ANOVA	Analysis of variance
HSD	Honestly Significant Difference
BF	Bright field
FB	Field blank
MB	Method (laboratory) blank
