# Supplementary material for: Microplastics in Stormwater: Sampling and Methodology Challenges
Source: Toxics. 2025 Jun 14;13(6):502. doi: 10.3390/toxics13060502 (PMC12197676; doi:10.3390/toxics13060502)
Supplement: Supplementary file 1 [file toxics-13-00502-s001.zip › toxics-3635853-supplementary.pdf]

SUPPLEMENTARY INFORMATION FOR:

Microplastics in Stormwater: Sampling and Methodology Challenges

Andres Sanchez Garcia [1], Huayun Zhou [2], Cesar Gomez-Avila [1], Tariq Hussain [3], Aryan Roghani [4], Danny Reible [1,2], and Balaji Anandha Rao [2*]

1 Department of Chemical Engineering, Texas Tech University, Lubbock, TX 79409, USA; andres.sanchez@ttu.edu (A.S.G.)
2 Department of Civil, Environmental & Construction Engineering, Texas Tech University, Lubbock, TX 79409, USA
3 Haley & Aldrich, Portland, OR 97239, USA
4 Department of Biomedical Engineering, University of Texas at Austin, Austin, TX 78712, USA
* Correspondence: balaji.rao@ttu.edu; Tel.: +1-806-392-5315

The impact of poorly cleaned instruments and how contamination can be carried by it has been previously reported (Hidalgo-Ruz et al., 2012). In figure S1, the effect of rinsing pipette tips shows a significant reduction in contamination of particles. Even though not all the particles identified by visual microscopy are actual MPs, they are likely to be counted during visual microscopy analysis.

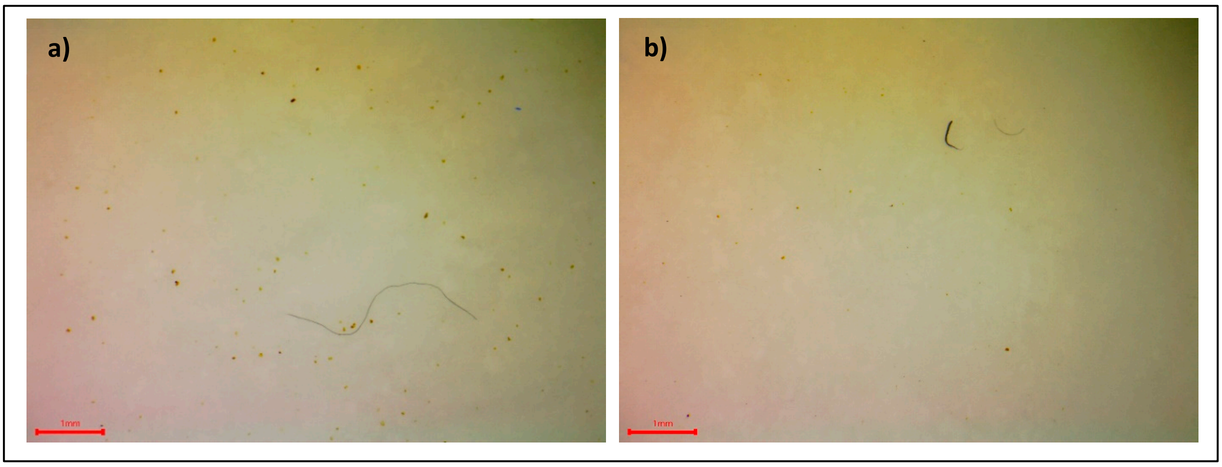

Figure S1 *Filter treated with a) an un-rinsed pipette tip and b) a rinsed pipette tip*

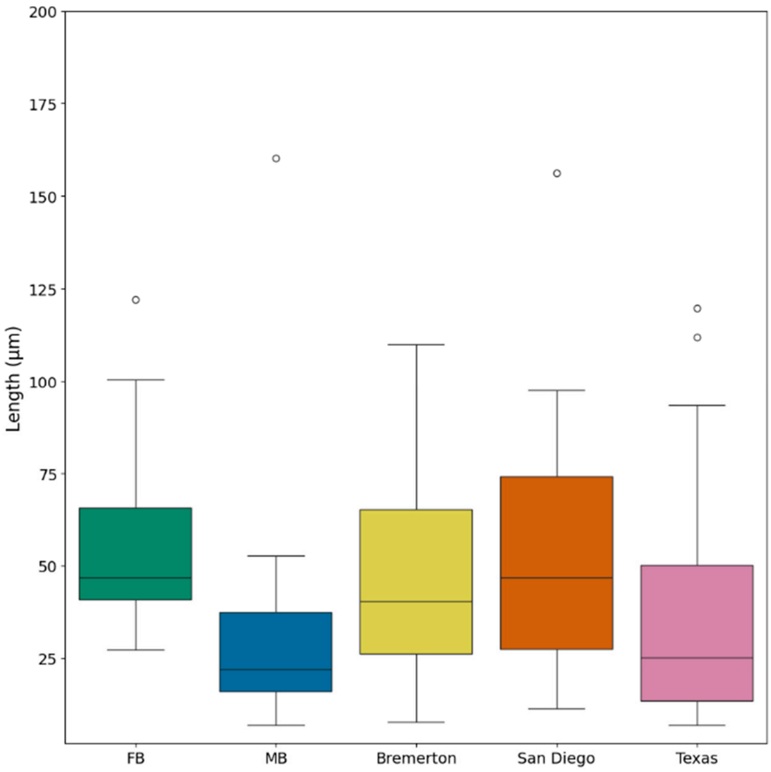

Figure S2. Particle size distribution for each location from visual analysis results.

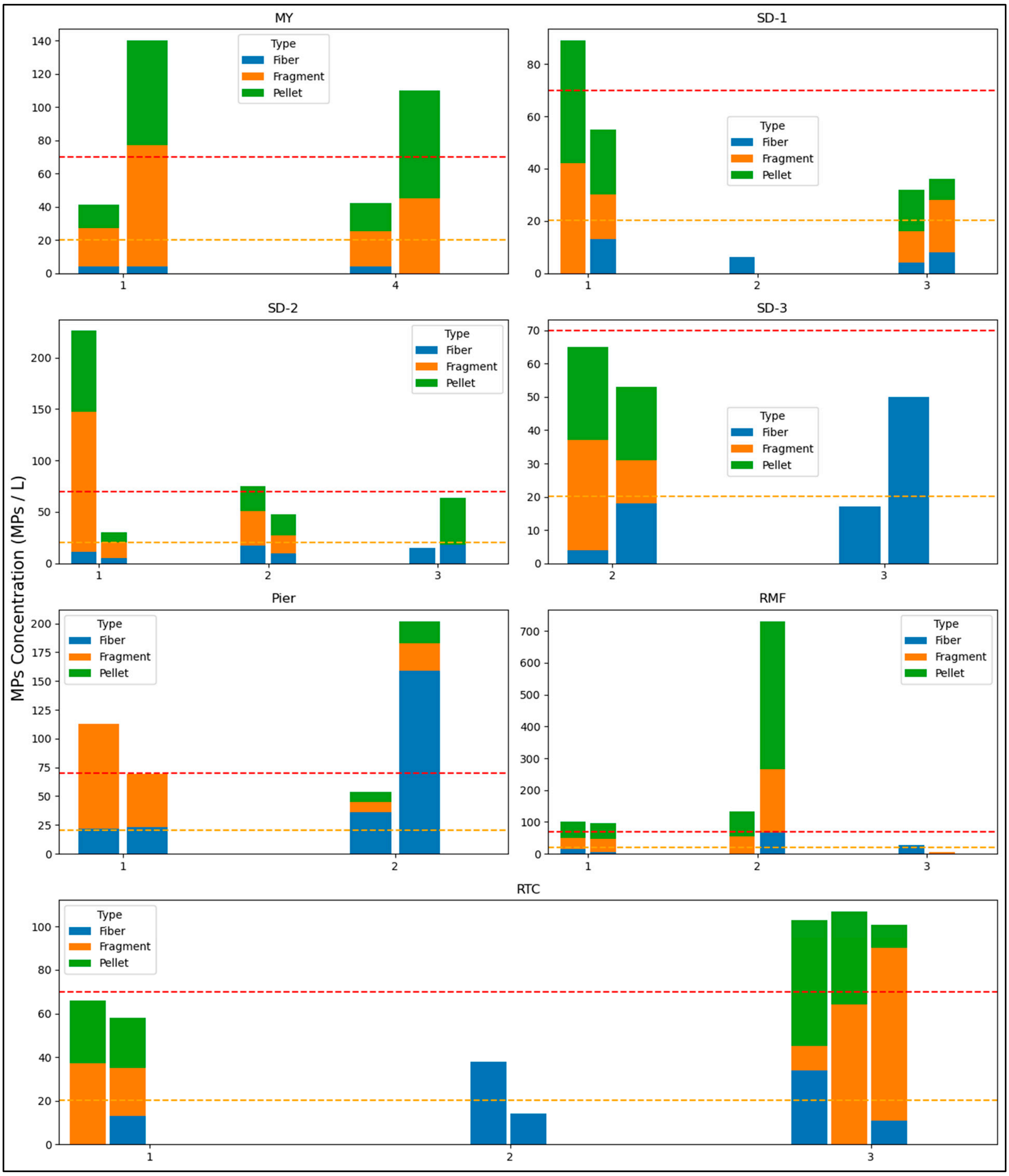

Figure S3 Results from the Visual Analysis for each site and rain event where data was available. Each pair of bars represents Inlet (left) and Outlet (right) of the SCM for each event, except for RTC where Inlet 1, Inlet 2 and Inlet 3 are compared. The horizontal lines represent the total concentration of MPs estimated in the laboratory blanks (orange) and the field blanks (red) with values of 24 and 71 MPs/L, respectively.

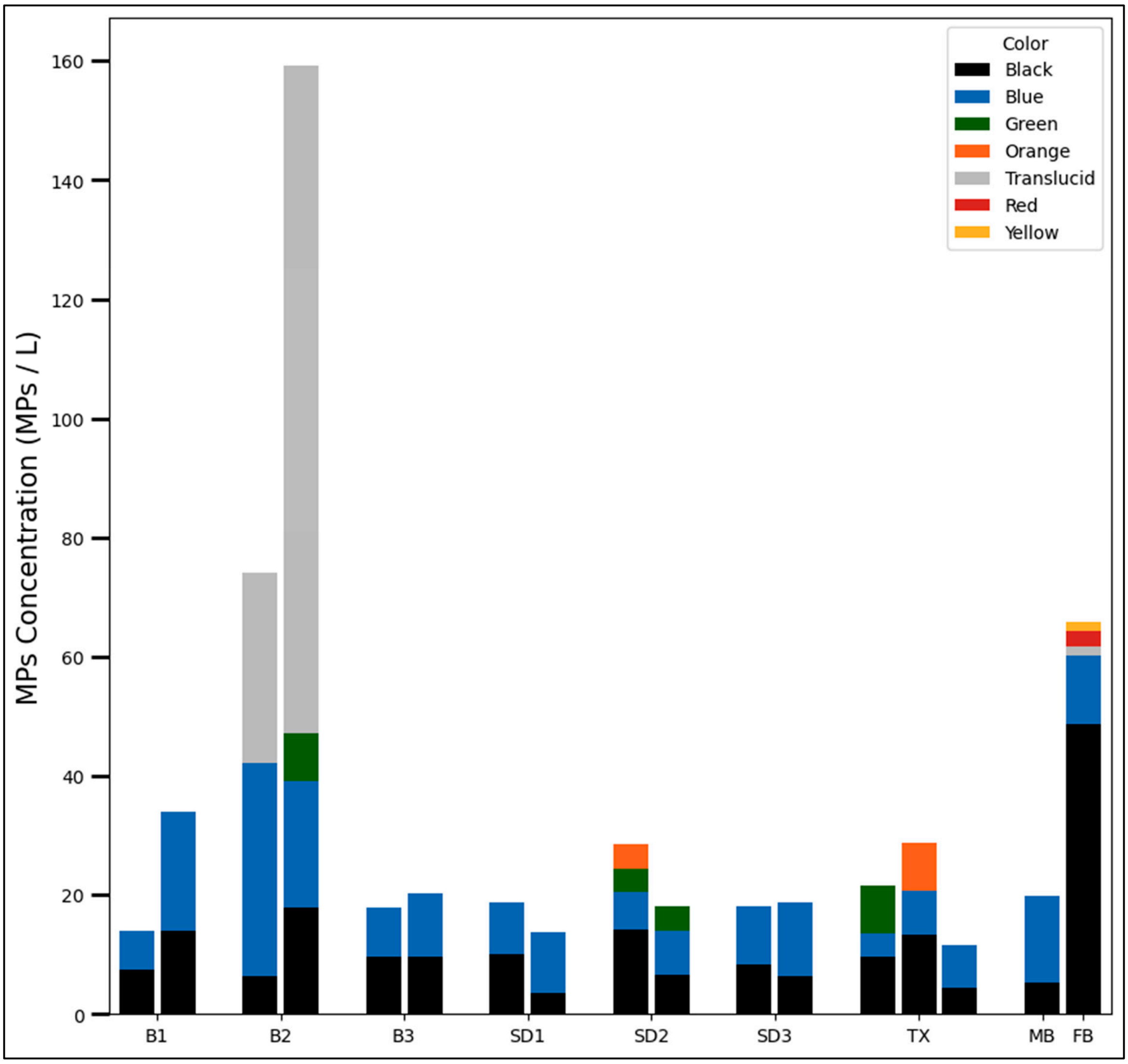

Figure S4. Results from visual analysis on terms of MPs color for each site. Inlet (left bar) vs Outlet (right bar) of the corresponding SCM is displayed for each location, except for TX where Inlet 1, Inlet 2 and Inlet 3 are compared. The values displayed correspond to the geometric mean of rain events per sample. Differences in the grouping used to calculate the geometric mean create discrepancies in the total concentrations when compared to those on Table 2.

Table S1. Raw particle counting from visual microscopy analysis. Values correspond to the geometrical mean observed on an AlOx filter.

Site	Fiber	Fragment	Pellet	Total
**B1 Inlet**	1.0	2.0	2.1	5.1
**B1 Outlet**	1.0	4.1	6.4	11.5
**B2 Inlet**	3.2	6.3	2.0	11.5
**B2 Outlet**	5.4	7.1	4.0	16.5
**B3 Inlet**	1.7	2.1	3.1	6.9
**B3 Outlet**	1.0	2.4	5.3	8.7
**SD1 Inlet**	1.0	2.7	3.0	6.7
**SD1 Outlet**	2.4	1.6	1.7	5.7
**SD2 Inlet**	1.3	6.1	3.1	10.5
**SD2 Outlet**	1.4	1.8	1.6	4.8
**SD3 Inlet**	1.4	3.5	3.0	7.9
**SD3 Outlet**	3.6	1.4	2.0	7.0
**TX Inlet 1**	1.4	2.1	5.9	9.4
**TX Inlet 2**	2.2	2.1	4.5	8.8
**TX Inlet 3**	1.7	2.2	1.0	4.9
**MB**	1.3	2.3	1.7	5.3
**FB**	3.4	13.0	0.0	16.4

Table S2. Samples that were identified manually as ‘quick compare’ function fail to automatically identify them. The orange spectra in each graph corresponds to the standard while the blue spectra come from the actual sample.

Microscope image	Spectrums
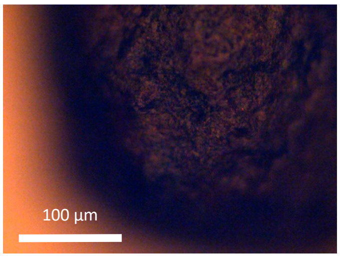	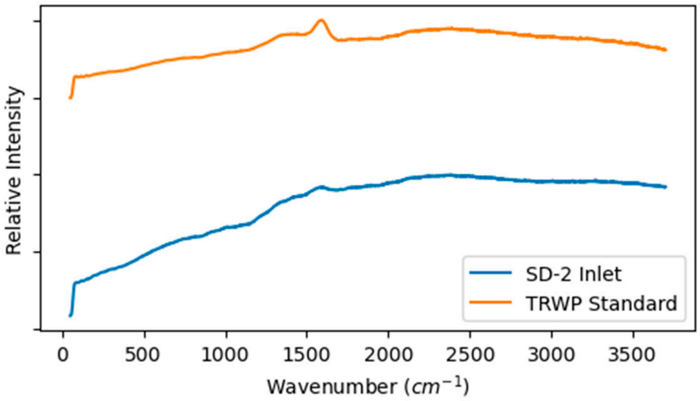
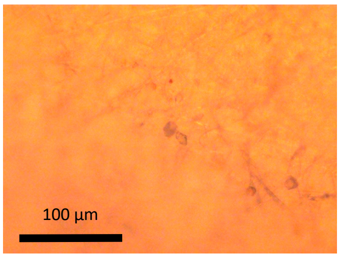	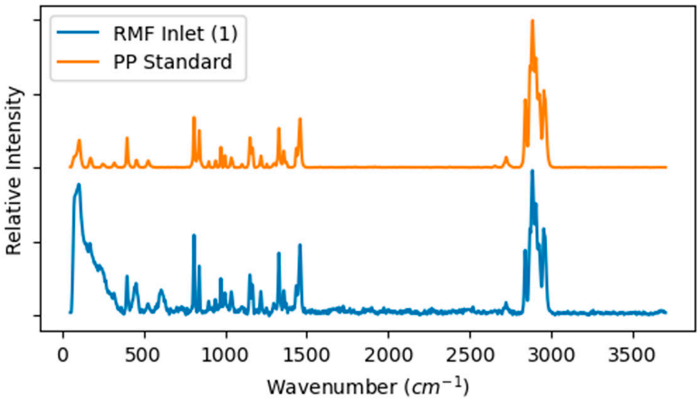
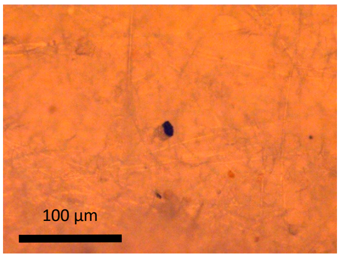	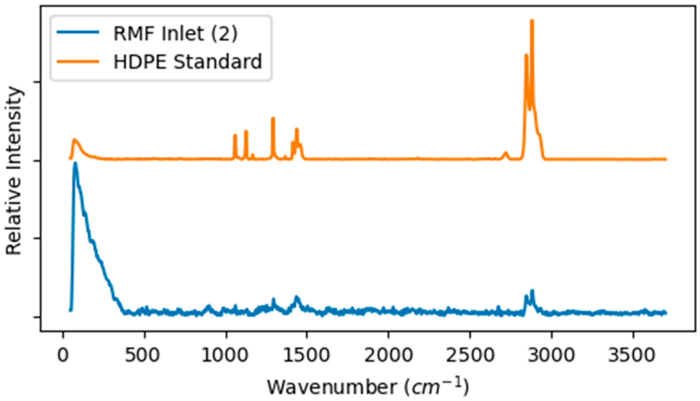

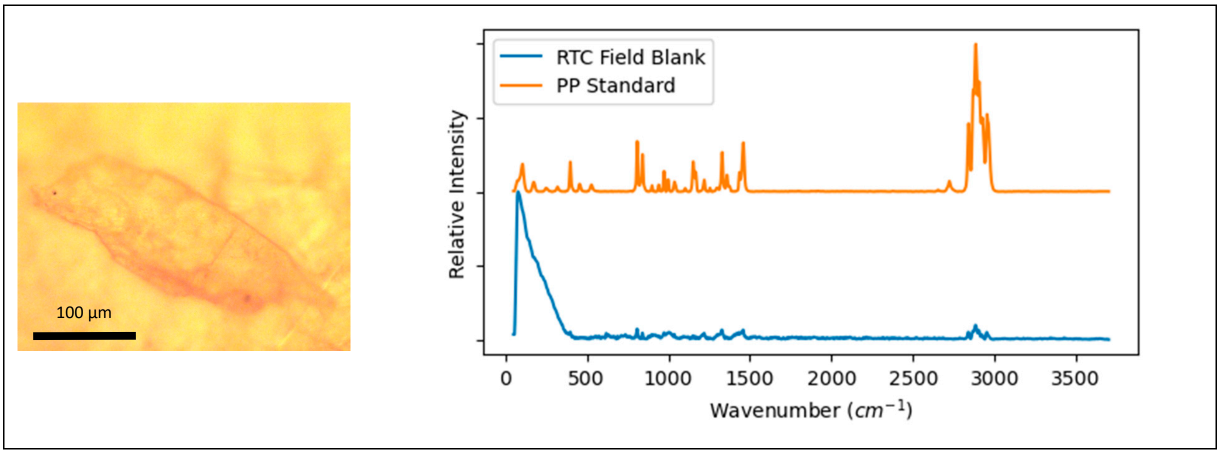
100 µm
RTC Field Blank
PP Standard
Relative Intensity
0
500
1000
1500
2000
2500
3000
3500
Wavenumber (cm^{-1})
